# Uses of Natural Honey in Cancer: An Updated Review

**DOI:** 10.34172/apb.2022.026

**Published:** 2021-02-01

**Authors:** Tahereh Eteraf-Oskouei, Moslem Najafi

**Affiliations:** ^1^Department of Pharmacology and Toxicology, Faculty of Pharmacy, Tabriz University of Medical Sciences, Tabriz, Iran.; ^2^Medical Education Research Center, Health Management and Safety Promotion Research Institute, Tabriz University of Medical Sciences, Tabriz, Iran.

**Keywords:** Natural honey, Human diseases, Cancer prevention, Cancer treatment

## Abstract

Cancer was predicted as the leading cause of death and the most important obstacle to the increased life expectancy in the 21st century worldwide. The World Health Organization (WHO) estimated number of new cases of cancers in 2020 about 19 million, and this number is estimated to be more than 295300000 people up to 2040 (more than 55% increase during next 20 years). Standard treatments for cancer include surgery, radiotherapy, and chemotherapy. However, all of these methods have dangerous side effects, so researchers are more interested in finding novel and less risky therapies. In recent years, there has been a great deal of interest in the development of anticancer agents obtained from foods or natural products. The relative safety of natural and food-derived compounds makes them attractive alternatives to conventional cancer treatment drugs. As a result, the majority of people are advised to use complementary and alternative medicine to treat and prevent cancer. In recent years, honey, as a natural product, has attracted many researchers’ attention as an alternative to conventional anticancer drugs. Natural honey has long been used as a medicine and nutrient and its beneficial effects on various diseases in animal and human models have been studied. It was found that it has a wide range of therapeutic properties, including antioxidant, antibacterial, antiviral, anti-fungal, anti-diabetic, anti-inflammatory, anti-hypertensive, antiarrhythmic, wound healing, and liver protection benefits. This article aimed to review the role of natural honey in the prevention and treatment of a number of important cancers and their subsequent complications.

## Cancer disease

### Introduction

Predictions suggested cancer as the most important cause of mortality^1-3^ and the major obstacle to the increased life expectancy in the 21st century.^1^ In 2018, there were 18.1 million new identified cancer cases and 9.6 million cancer-related deaths.^1^ The World Health Organization (WHO) estimated number of new cases of cancers in 2020 about 19 million, and this number will reach more than 295 300 000 people up to 2040 (more than 55% increase during next 20 years).^1^

### Definition and stages of cancer

Cancer is a disease characterized by deficiencies in the natural control mechanisms ruling cell survival, proliferation, and cell differentiation.^3,4^ The process of cancer progression consists of the following three basic stages: initiation, promotion, and progression.^3,5^ The first stage of cancer is associated with irreversible genetic damage, which is characterized by the accumulation of mutated DNA. The second stage (promotion) is resulted from overgrowth of mutant cells and further genomic alterations, causing benign masses of abnormal cells as tumors. Finally, the development or spread of cancer cells during metastasis process to distant sites through the lymphatic or circulatory systems is known as the third stage (progression).^3^ This dynamic process is activated by various carcinogens, tumor promoters, and inflammatory factors. Accordingly, this whole process is also controlled by transcription factors, proapoptotic proteins, anti-apoptotic proteins, protein kinase enzymes, cell cycle proteins, cell adhesion molecules, cyclooxygenase-2 (COX-2), and other molecular targets.^6^

### Causes of cancer

**Figure 1:**
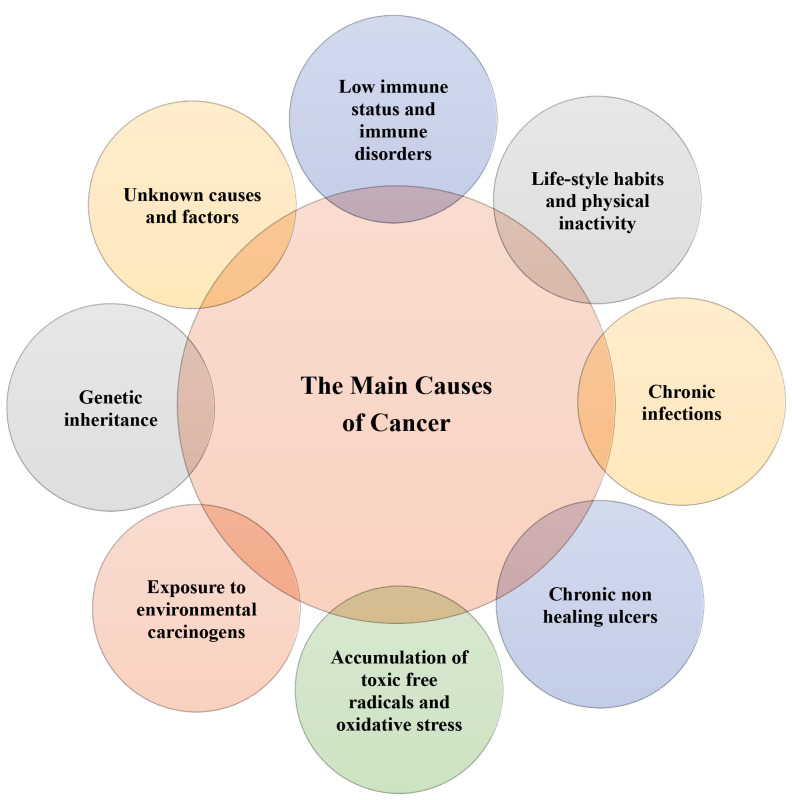
Schematic summary of cancer causes.

The incidence of different cancers, their specific behavior, and geographical distributions are related to several factors such as race, genetic characteristics, gender, age, and exposure to environmental carcinogens.^4^ Schematic summary of cancer causes is depicted in Figure 1. In this regard, the causes of cancer can be categorized as follows:

**A.** Low immune status due to having diabetes (especially type 2), chronic disease, aging, and obesity: Patients with low immune systems are more at the risk of cancer. This suggests why people with diabetes are at higher risk of epithelial cancers compared to people without it. Patients infected with human immunodeficiency virus (HIV) are also at the increased risk of developing epithelial and non-epithelial cancers and chronic infections, indicating a multiplicity of cancers. Patients with autoimmune diseases are also at the increased risk of colorectal cancer (CRC) in people with ulcerative colitis, Crohn’s disease, and thyroid cancer in autoimmune thyroiditis.^5,7^

Although older age alone is not known as an important determinant of cancer risk, it indicates long-term exposure to carcinogens. Aging is also associated with weaker immune system, which can be recognized as a risk factor for cancer.^5^

Obesity is closely linked to diabetes^5^ and in a society with a high prevalence of obesity, the prevalence of diabetes would also be high.^8^ One of the most common cancers under these conditions is CRC. For example, a study on 138 patients with CRC in Malaysia found that 47.8% of them had metabolic diseases, of which 13.8% were related to type 2 diabetes.^5^

**B.** Life-style habits and physical inactivity: Overall, one-third of cancers are due to smoking, one-third to people’s diet and lifestyle, and one-fifth of them are due to infections. Other factors include carcinogens, environmental pollutants, and alcohol.^5^ Tobacco smoking is associated with a number of cancers such as larynx, bladder, breast, esophagus, and cervix cancers. Accordingly, smoking increases the risk of CRC up to 43%.^5^ The risk of cancer in obese people is about 1.5 to 3.5 times higher than that of people with normal weight. In addition, obesity is associated with a number of cancers, especially endometrium, breast, and CRC.^5^

**C.** Chronic infections:Chronic infections caused by various pathogens, including *Helicobacter pylori* (gastric cancer),^5,7^ *Ureaplasmaurealyticum* (human prostate cancer),^5^ humanpapillomavirus (cervical, skin, and penis cancers),^4,5,7^ Epstein-Barr virus (nasopharyngeal carcinoma),^5,7^ Burkitt’s lymphoma, Hodgkin’s lymphoma^4^, hepatitis B and C viruses (hepatocellular carcinoma; HCC),^4,5,7^ HIV (Hodgkin’s and non-Hodgkin’s lymphoma),^4^ Schistosomiasis (bladder cancer), and *Aspergillus flavus* (HCC), have been implicated in cancer.^7^

**D.** Chronic non-healing ulcers:Chronic wounds would also increase the risk of cancer.^5^ Marjolin’s ulcer is the most common non-healing ulcer that presents in developing countries, especially in rural areas with poor living conditions. Correspondingly, this risk factor is associated with chronic infections and most of these wounds cannot be healed due to persistent infection.^5^

**E.** Accumulation of toxic free radicals and oxidative stress is due to alcohol consumption, obesity, smoking, and chronic inflammatory processes.^7^

**F.** Exposure to environmental carcinogens:Exposure to carcinogens in the environment is probably the most important cause of cancer. Having contact with ionizing radiation also is another important and proven risk factor for a number of cancers, including acute leukemia, soft tissue sarcoma, lung cancer, thyroid, breast, basal, and squamous cell skin carcinomas. The role of chemical carcinogens (especially those found in tobacco smoking) as well as aflatoxins, benzene, asbestos, radon, and azo dyes have been well demonstrated in many human cancers.^4^

**G.** Genetic inheritance: Cancer is caused by genetic damage to the cell’s genome. The damage either is genetically inherited or is acquired during life. Notably, acquired genetic damage is often imposed through unhealthy lifestyles.^7^

H. Unknown causes and factors^7^

### Treatment of cancer

The rapid development of human knowledge in the field of cancer biology and by analyzing cancer cell genome changes in thousands of samples obtained from patients have led to a better understanding on the molecular evolution of cancer and the discovery of specific therapeutic targets for cancer treatment such as intracellular signaling pathways, cell death pathways, growth factor receptors, tumor vascularity, epigenetic processes, defects in DNA repair, and immune system escape mechanisms.^9^

Surgery, radiotherapy, and chemotherapy are the standard treatments for cancer; however, all of these procedures have serious side effects yet.^6^ Treatment of cancer using chemotherapy drugs along with applying the current methods is associated with multidrugs’ resistance and several side effects.^2,3,9-11^ Besides these restrictions, availability of chemotherapy drugs and having access to them in some parts of the world (especially in developing countries) still is not easy.^3^ So, scientists have been attracted towards the development of some alternative, less toxic, and novel treatment methods against cancer.^2,11^

The relative safety of food-derived compounds makes them attractive alternatives to conventional anticancer drugs.^2^ As a result, the majority of people also prefer using complementary and alternative medicine (CAM) to treat their diseases.^3^ Recently, honey, as a natural compound, has attracted many researchers’ attention as a CAM.^10^ This article aimed to review the key roles of honey in the prevention and treatment of major cancers.

## Natural honey

### Types of natural honey

There are about 320 known different types of honey worldwide made from different sources of flowers. The flavor, color, and odor of each type of honey depends on the flowers and plants used by the bee to make it.^12^

### Physical properties of natural honey

In addition to its composition and taste, honey has several other important qualitative characteristics. Freshly extracted natural honey is a viscous liquid, viscosity of which depends on many substances such as honey compounds and especially on the water content. Hygroscopicity (the ability to absorb and retain ambient moisture) is another physical property of honey. It is noteworthy that honey with 18.8% or less water content absorbs moisture from the environment with a relative humidity above 60%. The color of liquid honey ranges from colorless to dark amber or black and varies depending on the plant’s origin, age, and storage conditions, but its transparency or clarity mostly depends on the content of the suspended particles.^13^

### Chemical constituents of natural honey

**Figure 2:**
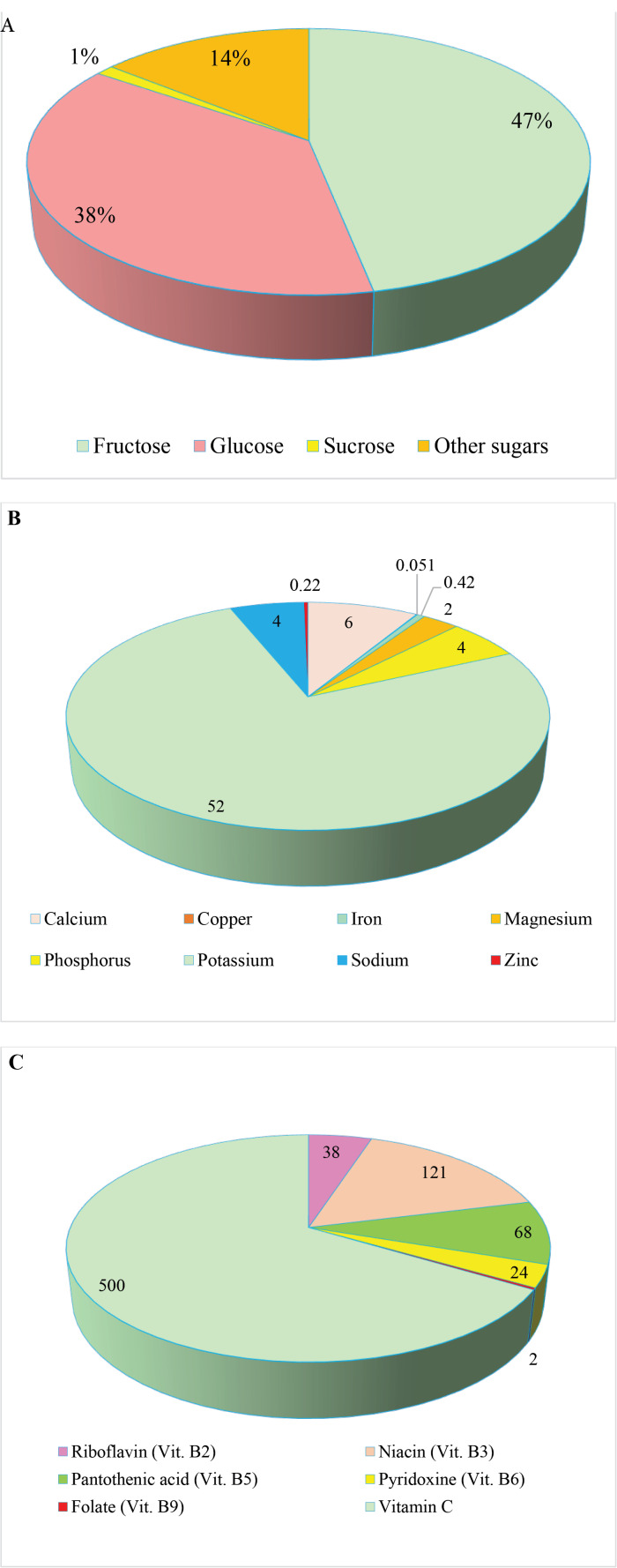
The average composition of natural honey. (A) Carbohydrates (percent/100 g), (B) Important minerals (mg/100 g), and (C) Important vitamins (μg/100 g).

The chemical composition of natural honey varies depending on the floral source used by the bee. The average composition of natural honey is presented in Figure 2 (A, B, C).^14,15^

Natural honey is composed of more than 200 different substances, including amino acids, vitamins, minerals, and enzymes.^2,10,11,14^ However, its major components are sugar and water. Accordingly, sugars in honey make up about 95-99% of its dry matter.^14^ Carbohydrates are the other main chemicals of honey, which comprise two major monosaccharides as fructose (32.56 to 38.2%) and glucose (28.54 to 31.3%), along with small amounts of 22 other types of sugar. The identified disaccharides in honey include maltose, sucrose, maltulose, isomaltose, laminaribiose, turanose, kojibiose, nigerose, gentiobiose, and B-trehalose.^2,10,11,14^ Trisaccharides include maltotriose, isomaltotriose, melezitose, panose, isopanose, centose 3-a5, erlose, isomaltosylglucose, l-kestose, and theanderose.^10,14^ Moreover, there are proteins, pigments, volatile compounds,^2,11^ flavonoids, and phenolic acids in honey composition.^2,10,11,14^ Twenty six types of amino acids have been reported in honey, of which proline accounts for 50-85% of total amino acids exist in honey.^10^ Minor amounts of vitamins include thiamine,^2^ niacin, riboflavin, pyridoxine, ascorbic acid, folic acid, nicotinic acid, and pantothenic acid.^14^ Notably, potassium is the main metal found in honey, followed by calcium, magnesium, sodium, sulfur, and phosphorus, respectively.^10,11,14^

Organic acids in natural honey are as found: acetic, butyric, gluconic, lactic, citric, succinic, malic acid, and a number of other aromatic acids.^10,14^ Some of flavonoids and phenolic compounds identified in honey are the followings: quercetin, chrysin, kaempferol, myricetin, galangin, pinobanksin, luteolin, apigenin, genistein, pinocembrin, hesperetin, p-coumaric acid, naringenin, ferulic acid, syringic acid, gallic acid, caffeic acid, ellagic acid, and vanillic acid.^2,3,10,11^

### Enzymes

Different enzymes found in natural honey are glucose oxidase, diastase (amylase), invertase, superoxide dismutase (SOD), catalase, alpha-glucosidase, protease, esterase, and acid phosphatase.^3,10,14,16^ However, the major enzymes exist in honey are known to be glucose oxidase, invertase, and diastase.^14^

### Pharmacokinetics of natural honey

No reports on the pharmacokinetics of natural honey were found in the literature. However, some experimental and clinical studies have revealed that pharmacokinetic parameters of some drugs may be altered by honey.^10^ For example, plasma concentrations of intravenously and orally administered diltiazem (a calcium channel blocker) have decreased by natural honey in rabbits.^17^ Results of another animal study by Koumaravelou et alhave demonstrated that single and multiple doses of honey could decrease the bioavailability of carbamazepine.^18^ Activity of cytochrome P450 2C8 (CYP2C8) was dose-dependently inhibited by Tualang honey (TLH) under *in vitro*. This effect may cause drug-food interactions with medications metabolized via CYP2C8 enzymes.^19^

Results of some human studies have shown that natural honey interferes with the activity of some CYP450 isoenzymes. Moreover, it was shown that regular consumption of honey may increase the activity of CYP3A4 without altering the activity of any other enzyme such as CYP2D6 or CYP2C19. Therefore, honey may induce the altered responses to drugs metabolized by CYP3A4. In contrast, another clinical study reported that daily consumption of honey did not affect the activity of CYP3A enzymes and P-glycoprotein.^10^

## Therapeutic effects of natural honey

### 1. Therapeutic uses in non-cancer diseases

Natural honey has long been used as a food and also as a medicine.^11^ Accordingly, its effects on various diseases in animal and human models have been studied.^10^ Honey has a wide range of therapeutic properties such as antibacterial, antiviral, antifungal, antioxidant, anti-inflammatory, anti-angiogenic, wound healing, antidiabetic, hepatoprotective, antihypertensive, and antiarrhythmic properties.^3,10,11,20^

Natural honey has an inhibitory effect on about 60 species of aerobic and anaerobic, gram positive and gram negative bacteria such as *E. coli*, *Salmonella, Shigella*, *Vibrio cholerae, Bacillus anthracis, Helicobacter pylori*,*Corynebacterium diphtheriae, Klebsiella pneumoniae, Haemophilus influenzae, Proteus species, Pseudomonas aeruginosa*, *Mycobacterium tuberculosis, Listeria monocytogenes, Pasteurella multocida, Yersinia enterocolitica, Serratia marcescens, Acinetobacter*spp, *Staphylococcus aureus,*and* Streptococcus pyogenes*.^14^

One of the most useful uses of honey that has been extensively studied is its effect on wound healing. It appears to treat almost all types of ulcers such as abrasion, abscess, bed sores/decubitus ulcers, burns, chilblains, amputation, fistulas, nipple cracking, diabetic, malignant, and surgical wounds.^14^

Cardiovascular diseases are one of the most important diseases for which natural honey is used for prevention and treatment. In coronary heart disease (CHD), the protective effects of phenolic compounds present in honey include antioxidant, anti-ischemic, anti-thrombotic, and vasorelaxant functions. Flavonoids appear to reduce the risk of CHD by the following three main mechanisms: improving coronary artery dilation, lowering platelet ability to coagulate, and preventing the oxidation of low-density lipoproteins.^14^ Honey can also lower venous blood pressure, which can consequently reduce the preload of the heart and congestion of the venous system.^14^ Researchers have shown the prophylactic effects of natural honey as a preconditioning agent, on ischemia/reperfusion induced arrhythmias and infarct size in isolated rat’s heart.^21,22^ In addition, the results of another study showed that chronic oral administration of natural honey (45 days) has potent antiarrhythmic and anti-infarct effects on rats.^23^ Honey could also inhibit oxidative stress, which may be partly responsible for its neuroprotective activity in *in vitro* cell death and *in vivo* focal cerebral ischemia.^14^

Gastrointestinal infections such as gastritis, gastric and duodenum ulcers caused by bacteria and *rotavirus*have been treated and protected by oral honey. In addition, diarrhea and gastroenteritis are quickly alleviated by honey. In the management of gastric ulcer, honey has some healing properties and may be used in a manner similar to sucralfate.^14^

Honey is useful in neurological diseases because it exerts antidepressant, anxiolytic, antinociceptive, and anticonvulsant effects. It also modifies the oxidative content in the central nervous system.^24^

Natural honey is also used in the treatment of chest pain, vertigo, and fatigue. These effects can probably be due to the high energy content in honey, providing necessary calories immediately after its consumption. In a study, daily intake of honey showed various beneficial effects on enzymes, endocrine system, hematological indices, and blood mineral levels. Furthermore, it has a beneficial effect on immune system; for example, by antibody production in response to thymus-independent and thymus-dependent antigens.^14^

Honey has also been shown to reduce asthma-related symptoms and prevent asthma induction.^24^ In allergic rhinitis, it was found that the simultaneous consumption of honey (1 g/kg; 4 weeks) with the standard medications improves the overall symptoms of the disease.^25^

Preventing uterine atrophy, suppressing body weight gain, and increasing bone density in menopaused animals were demonstrated by honey, which could be used as an alternative to hormone’s replacement therapy in menopause.^14^ Honey intake can also be useful in men with impotence and infertile women who are infertile due to some reasons like erratic ovulation.^12^

Oral use of raw honey has positive effects on oral wellness and dental health, which can also be used during dental surgery. The use of Asian polyfloral honey reduces pain associated with tooth extraction and prevents oral infections and dental cares.^26^

Honey is used to treat various eye disorders such as conjunctivitis, keratitis, blepharitis, corneal injuries, and thermal and chemical burns. In 102 patients with non-responsive eye diseases, application of ophthalmic honey ointment improved these disorders in 85% patients and there was no disease’s progression in the rest of them.^14^

### 2. Honey as a potential cancer prevention agent

Negative side effects of chemotherapy drugs can affect the patients’ quality of life (QoL). Therefore, treatment modalities that can prevent the progression of malignancy, reduce the dose required by conventional anticancer drugs or decrease the severity of their adverse effects, are desirable with significant benefits.^2^

In a model of carcinogenesis in rats, Manuka honey (MKH) and TLH were administered 7 days before the induction of breast cancer with N-methyl-N-nitrosourea. Notably, tumor’s development was significantly inhibited where the effect of MKH was greater than TLH.^27^ The results of another study have also shown decrease in the grade, size, angiogenesis, and vascular endothelial growth factor levels in breast cancer induced by 7, 12-dimethylbenz (*a*) anthracene (DMBA).^28^ In a study, the effects of Iranian natural honey (from Oskou, East Azerbaijan) on DMBA-initiated and croton oil-promoted skin carcinogenesis were investigated in Albino Swiss mice. Firstly, topical honey was used several times in the animals as a pre-treatment and after 9 h, the cancer was induced by DMBA. Thereafter, the promoting agent (croton oil) was topically applied twice a week for a 30-week period. Honey-treated group showed a significant inhibition of tumor incidence compared to the control group. In addition, the enhanced uptake of [3H]-thymidine in mice skin DNA was inhibited in those animals pretreated with honey as compared to the control group. The authors concluded that croton oil-mediated tumor promotion is inhibited by the antioxidants exist in honey.^29^

Prophylactic effect of Coriander honey (CDH) on cancer in Ehrlich ascites carcinoma (EAC) model has been shown under both *in vitro*and* in vivo*. The administration of CDH to the animals lowered the volume of ascetic fluid and the number of viable tumor cells and also increased their lifespan. The decreased lipid peroxidation and SOD levels, as the glutathione levels, have increased after honey administration, indicating that the antitumor activity of CDH was due to its antioxidant effect.^30^ It was shown that the treatment of EAC cells with honey inhibits viability of tumor cells and cell proliferation, while honey pretreatment in EAC mice clearly diminishes tumor size. Prophylactic treatment with honey also increased the levels of bone marrow lymphocytes and peritoneal macrophages in these animals, indicating that honey strengthens the immune system by the activation of macrophages and T and B cells’ functions.^31^ The activation of the immune system by honey has been confirmed in another study, in which the levels of immunoglobulins M, G, and A have increased after the administration of honey in mice.^32^

Acacia honey (ACH) produced by *Apis mellifera* fed with acacia flowers has been reported to be able to cause an antiproliferative effect on melanoma cells.^33^ In addition, the preventive effects of individual active compounds extracted from honey on cancer have been demonstrated.^34^

### 3. The therapeutic effect of honey against cancer

In recent years, many studies have been conducted on the use of natural products to treat cancer. Among them, natural honey has been considered by many researchers. Correspondingly, its potential benefits to treat important cancers have been studied in experimental and clinical settings.

#### Breast cancer

Tsiapara et al in their study evaluated the effects of Greek thyme, fir, and pine honey extracts on the modulation of estrogenic activity and viability of MCF-7 breast cancer cells.^35^ They reported that the honey samples had biphasic behavior depending on their concentrations. Notably, the extracts exhibited antiestrogenic effect at low concentrations and estrogenic activity at high concentrations. Although in the presence of estradiol, pine and thyme honey (THH) exert antiestrogenic activity, fir honey extract increases estrogen activity in MCF-7 cells. Additionally, pine and THH had no effect on MCF-7 cell viability; however, fir honey increased the viability of these cells.^35^ These dual effects are likely due to the presence of high amounts of phenolic compounds such as quercetin and kaempferol, in the extracts. Many previous studies have reported such these dual actions by phenolic compounds and other phytoestrogens.^36^

TLH is a multifloral honey produced by Asian rock bees called *Apis dorsata*.^37^ Cytotoxic effects of TLH on human breast cancer cell lines, including MCF-7 and MDA-MB-231, has been shown^38^ to be along with the increased membrane lactate dehydrogenase leakage. TLH also induces apoptosis and reduces the mitochondrial membrane’s potential.^3,34^ In a study by Abd Kadir et al, the effect of TLH on DMBA-induced breast cancer development was investigated in rat. They found that tumor development begins much earlier in the untreated (control) animals compared to the honey-treated group.^28^

The apoptotic effects of tamoxifen on human breast cancer cell lines (ER- responsive and ER-nonresponsive cells) can be promoted by its co-administration with TLH.^39^ Compared to the administration of tamoxifen alone, combination of tamoxifen with TLH more increases total apoptosis in MCF-7 cell line. Analysis of flow cytometry data has also shown that apoptosis is the favorite mechanism of cell death, because the involvement of caspase 3, 7, 8, and 9 are observed under these conditions.^40^

Growth of MCF-7 breast cancer cell line is inhibited by ACH in a time- and dose- dependent manner.^34^ In an experimental model of breast cancer in mice, the antimetastatic activity of honey when used prior to the inoculation of tumor cells, has been reported.^3^ This effect may be due to the presence of flavonoids such as chrysin, in honey composition.^41^ In addition, the combination of *Aloe vera*and honey inhibits cell proliferation and suppresses tumor growth in Walker 256 carcinoma implanted in rats.^42^

#### Liver cancer

The results of a study showed that the treatment of human hepatocellular carcinoma (HepG2) cells with honey could clearly reduce nitric oxide levels and the number of viable HepG2 cells, while increasing total antioxidant status.^43^ Regarding these data, it seems that the survival of HepG2 cells depends on reactive oxygen species (ROS). Therefore, decreasing ROS and improving antioxidant defense by honey lead to the inhibition of proliferation and the decreased number of viable HepG2 cells.^3^ The results of a study by Baiomy et alin HepG2 cells showed that honey extracts have cytotoxic, anti-angiogenic, and antimetastatic activities. However, the intensity of these effects varies depending on the quality of the honey used.^44^

Gelam honey (GLH) is a Malaysian monofloral honey produced by *Apis mellifera* from *Melaluca* spp. Because of high amounts of polyphenols that exert antioxidant and free radical scavenging properties, they are useful in the prevention of cancer as well as some other diseases. The results of a study on the antiproliferative effects of GLH on HepG2 cells revealed that its 50% inhibitory concentration (IC50) was only 25% for these cells and 70% for normal human liver cells (WRL-68). The study has also found that GLH inhibits HepG2 cells proliferation and induces apoptosis.^45^ Another study performed in a HepG2 cell line, examined the effects of three floral honey samples (heather, rosemary, and heterofloral) on DNA’s strand breakage.^46^ Correspondingly, the results revealed protective effects of honey samples on DNA damage induced by dietary mutagens benzo(a)pyrene, N-nitrosopyrrolidine, 2-amino-1-methyl-6-phenyl-imidazo[4,5-b]pyridine, but not on damage induced by N-nitrosodimethylamine. The study has also shown a relationship between high phenolic content of honey and HepG2 protection. Moreover, antioxidant and free radical scavenging properties of honey are involved in its protective effects on mutagens-induced DNA damage in HepG2 cells.^46^ As shown in another study, development and progression of diethyl nitrosamine -induced liver cancer were examined in rats in the presence of honey.^47^ After six months, the liver of those untreated rats had various lesions, including edema, destruction of adipose tissue with the displacement of cell nuclei, inflammatory lymphocytic infiltration, the injured hepatocytes with hyperchromatic nuclei, the presence of neoplastic hepatocytes, stained nuclei for *p53,* and proliferating cell nuclear antigen expressions. However, these abnormalities have clearly reduced in the liver of those rats treated by honey.^47^

#### Colorectal cancer

Antiproliferative effects of Nenas and Gelam monofloral honeys on colon cancer were indicated in HT 29 cell lines. In addition, both honey samples dose-dependently induced DNA damage and suppressed H_2_O_2_-induced inflammation in colon cancer cells.^48^ In a study by Jaganathan and Mandal, the apoptotic effects of some raw honeys on colon cancer cell lines (HCT 15 and HT 29) were investigated. Their results confirmed the antiproliferative effect of honey that was previously reported by other researchers. Notably, this effect depends on the level of phenolic compositions present in honey.^49^ Studies performed by Oršolić et al in murine colon carcinoma model revealed that if honey is used before tumor cells inoculation, it would have antimetastatic effects.^50^ Similarly, it was indicated that in anaplastic colon adenocarcinoma of Y59 rats, honey exerts antimetastatic effect.^51^ Moreover, GLH in combination with aqueous extract of ginger enhances the therapeutic effect of 5-fluorourouracil (5-FU) on colon cancer cells (HCT-116).^52^ The synergism of GLH plus ginger extracts has also been confirmed in HT29 cell line. Altogether, ginger and GLH modulate Ras/ERK and PI3K/Akt signaling pathways, which may be involved in the early phases of CRC formation.^53^

#### Prostate cancer

Antiproliferative effects of ACH on prostate cancer cells (PC-3 cell line) was evaluated by Aliyu et al. Results of the 3-(4, 5-dimethylthiazol-2-yl)-2, 5-diphenyl tetrazolium bromide (MTT) test showed that IC50 values for PC-3 cancer cells and normal NIH/3T3 cells were 1.9 and 3.7%, respectively.^54^ Results of Tsiapara and colleagues’ study on viability of PC-3 cell lines have also indicated some considerable differences among the effects of various extracts of Greek fir, thyme, and pine honeys. Their results found that only THH markedly reduced the viability of the cells.^35^

#### Bladder cancer

Based on global cancer’ statistics, bladder carcinoma is more common in men than women, which is ranked as the 10th most common malignancy with an estimated 200 000 deaths and 550 000 new cases in 2018 worldwide.^1^

Proliferation of human bladder cancer cell lines, including RT4, T24, and 253J cells, as well as a murine bladder cancer cell line (MBT-2) was significantly inhibited by honey. In addition, the effect of honey was studied in the *in vivo*model of bladder cancer. The results showed that intralesional and oral administrations of honey (6% and 12%) markedly suppressed tumor growth.^55^

#### Pancreatic cancer

Pancreatic cancer is known as the 7th deadliest cancer in both men and women with around 432 000 deaths in 2018 worldwide.^1^

Results of a study by Angst et alrevealed that oral administration of quercetin (a phenolic compound and the important flavonoid found in natural honey) inhibited the growth of human pancreatic cancer cell lines (MIA PaCa-2 and BxPC-3) under *in vivo* and *in vitro*.^56^ Furthermore, Naringenin (as a natural flavanone identified in honey), with a similar structure to chrysin, reduces metastasis and invasion in pancreatic cancer cell lines (Aspc-1 and panc-1). In addition, it was found that naringenin could reverse the TGF-b1-induced resistance to gemcitabine.^57^

#### Lung cancer

In a study by Aliyu et al, the potential anticancer activity of ACH was investigated in a lung cancer cell line (NCI-H460). Their results showed that low concentrations (2-4%) of ACH were unable to inhibit NCI-H460 proliferation. Moreover, TNF-α and IL-1β levels were also low under this condition, while these cytokines markedly increased at higher ACH concentrations. The researchers have attributed the observed effects to the apoptotic activity of ACH in NCI-H460 cell line and have also found that gene expressions of *p53* and *Bcl2* were down-regulated by ACH proportional to its dose used.^58^

#### Melanoma

Pichichero et alevaluated the effect of ACH on melanoma in human A375 and B16-F1 murine melanoma cells. Their results showed that honey could affect the metabolic activity of both studied cells in a dose- and time-dependent manner.^33^

Antitumor properties of MKH on melanoma were also investigated by Cabezudo et alin a mouse model. Based on their results, MKH could inhibit the growth of B16-F melanoma cells in a time- and dose-dependent fashion. In these cells, honey-induced apoptosis was mediated by the caspase-3 activation with a concurrent decrease in *Bcl2* and stimulation of DNA fragmentation.^59^

#### Renal carcinoma

In 2018, more than 403 000 new cases of kidney cancer were diagnosed and 175 098 patients have died because of this disease worldwide.^1^

To determine the effect of honey on renal carcinoma, human renal carcinoma cells were used. MTT assay results clearly showed dose- and time-dependent antiproliferative nature of honey on the above-mentioned cells. Also, a high concentration of honey (20%) showed shrinkage and structural changes of the cells, which are known as the characteristics of apoptosis.^60^

In a study by Song et al, the effects of Kaempferol (another important flavonoid of natural honey) on the growth of human renal cell carcinoma (RCC) were investigated. They reported the inhibition of cell growth, induction of apoptosis, and cell cycle arrest in kaempferol-treated RCC cells (786-O and 769-P cells). In addition, the activity of the EGFR/p38 signaling pathways was strongly inhibited by kaempferol.^61^

#### Leukemia

Antiproliferative and apoptotic effects of three samples of Spanish raw honey (rosemary, heather, and heterofloral) in human peripheral blood promyelocytic leukemia cell line (HL-60) were studied by Morales and Haza. They reported time- and concentration-dependent induction of apoptosis by the use of the samples. In addition, the apoptotic effect was shown to be dependent on the phenolic content of the honey samples.^62^ In acute and chronic K562 and MV4-11 leukemia cell lines, their proliferations were inhibited by TLH in a time- and dose-dependent manner. After 24 h treatment with TLH, an increase was observed in both early and late apoptosis in the cells.^63^

#### Other types of cancer

In many other types of cancer such as non-small cell lung cancer, cervical cancer, endometrial cancer, osteosarcoma, and oral squamous cell carcinoma (OSCC) cells, honey induces apoptosis, inhibits cell proliferation, changes cell cycle, and depolarizes mitochondrial membrane.^3^ Ghashm et al reported that different concentrations of TLH (1–20%) are effective on the treatment of OSCC.^64^ The antitumor activity of two honey samples with high and low phenolic contents against EAC has been demonstrated by Jaganathan et al. They found that the type of honey rich in phenolic compounds is able to inhibit EAC growth in comparison with the other varieties. Intraperitoneal administration of honey (25%) has also resulted in maximal inhibition of the tumor growth by approximately 40%.^65^ Honey produces moderate antitumor and considerable antimetastatic activities on different types of tumors in mouse and rat. In addition, it facilitates antitumor activities of cyclophosphamide and 5-FU.^66^

Fukuda et al evaluated antitumor and immune functions of a Nigerian jungle honey sample in female C57BL/6 mice. Peritoneal cells were obtained from the animals following daily injection of 1mg honey/mouse for a 7-day period. It was observed that the number of peritoneal cells has increased in the honey-treated group. Neutrophils were identified in honey-treated group by flow cytometry analysis. Therefore, they concluded that the jungle honey has chemotaxis for neutrophils. Furthermore, the incidence and weight of tumors have diminished in honey-receiving mice.^67^

### 4. Other effects of honey on cancer and cancer-related complications

The results of cell culture studies have shown that the cytotoxic effect of 4-hydroxytamoxifen was diminished by TLH in a non-tumor breast cell line (MCF-10A). While it enhances the anticancer activity of tamoxifen in breast cancer cells (MCF-7 and MDA-MB-231). Accordingly, this indicates that TLH augments tamoxifen effects on cancer cells, which in turn, protects healthy cells from its toxicity.^40,68^ In another study, C57BL/6 mice were divided into three groups receiving intravenous injection of 50% MKH suspension, 10 mg/kg paclitaxel or their combination twice a week. On days 20 and 24 after the treatment, caspase-3 immunohistochemical assay was performed to identify the number of apoptotic cells in the tumors. The results show higher caspase-3-positive cells in the combination-treated mice. Correspondingly, this suggests that MKH in combination with paclitaxel may reduce its cytotoxicity.^59^ In another study, the administration of a bee products’ mixture (including royal jelly, pollen grains, and honey) improved the genotoxic effects of cyclophosphamide in mice.^69^ Besides surgery, combination of radiotherapy with chemotherapy (especially cisplatin) is considered as the most common and gold standard for head and neck cancers’ treatment.^70^ However, this combination often leads to oral mucositis (OM),^71^ which affects almost 50% of patients,^34^ and its management still remains challenging.^2^

The beneficial effect of natural honey prepared from the Zagros Mountains in western Iran on chemotherapy induced OM has been reported in a randomized clinical trial. The results of this double-blind study revealed that the combination of honey and coffee was a healthier treatment for OM in comparison with topical corticosteroids.^72^ In a study performed in Kerman (Iran) on 105 patients with head and neck cancers undergoing radiotherapy, the administration of pure natural honey mouthwash was effective on preventing radiation-induced OM.^73^ In order to provide evidence for the effectiveness of THH on the radiation-induced OM, a randomized controlled trial study was performed on 72 patients with head and neck cancers. The results show that the patients in the intervention group (THH rinses) had a lower OM grade, better global health status, and higher QoL that was statistically significantly compared to the patients in the control group (saline rinses).^74^ Compared to betadine (povidone-iodine), honey decreased OM, incidence of intolerable mucositis, and the loss of treatment days in patients with head and neck cancers requiring radiotherapy. In addition, honey did not interfere with the anti-tumor effects of radiation therapy.^75^ Another clinical trial conducted by Jayalekshmi et al in 2016 showed that natural honey has a beneficial effect on OM of patients with head and neck cancers receiving radiotherapy.^76^

In 2017, the results of a clinical trial conducted by Amanat et al have also indicated that Ziziphus honey could significantly lower the severity of radiotherapy-induced mucositis in patients with head and neck cancers.^77^ However, in a double-blind clinical trial, researchers in Canada found that MKH was not well-tolerated by their patients. Moreover, even when applied directly on the oral lesions, it did not have a significant effect on reducing the severity of OM resulted from radiation therapy.^78^

Yang et al in a systematic review and network meta-analysis showed that honey is a safe and effective adjuvant treatment for OM-induced by chemotherapy and radiotherapy in cancer patients. Especially, the topical application of pure natural honey may be considered as a first-line adjuvant under this condition.^79^ The results of systematic reviews by Song et al^80^ and Van Den Wyngaer^81^ have shown the beneficial effects of honey on mucositis. However, in order to confirm its benefits, more clinical trials are needed. In addition, data obtained from a systematic review by Münstedt et al in 2019 indicated that unlike MKH, conventional honey is useful in the prevention and treatment of OM caused by chemotherapy and radiation therapy. Therefore, one of the reasons for the difference in test results in this field is the type of honey used.^70^

Another consequence of radiation therapy in patients with head and neck cancers is xerostomia (dry mouth).^34^ In order to assess the effectiveness of THH under this condition, 72 patients undergoing radiotherapy or/and chemotherapy were studied. The findings showed the safety and efficacy of THH on the management of xerostomia in these patients.^82^ In addition, benefits of honey in tooth decay, extraction pain, and infection related to radiotherapy-induced xerostomia, have been shown.^14^

The oral consumption of raw honey one week before up to three days after the cisplatin administration reduced its nephrotoxicity in rats. The mechanism of this protective function may be related to the suppression of nuclear factor kappa B (NF-κB) activity by honey.^83^ In another study, the administration of 20 mg/kg of honey for a 10-week duration decreased the serum levels of renal injury indicators (such as urea, creatinine, and uric acid) and histopathological changes to normal values in rats.^84^ In addition, the results of some studies performed in patients with cancer suggest that crude honey reduces renal toxicity of cisplatin. For instance, the patients who have daily consumed 80 g honey in their diet before and during cisplatin, were found to have lower serum creatinine and urea levels compared to the control group.^85^

In postmenopausal women with breast cancer who were treated with tamoxifen and aromatase inhibitors, daily consumption of a tablespoon of sunflower honey for a 2-week period decreased menopausal complaints. However, in the group receiving aromatase enzyme inhibitors, an increase was observed in estrogen levels, and this has consequently raised concerns on the use of honey for patients receiving these drugs.^86^

Cancer-related fatigue occurs in 50-90% of patients with cancer, which can affect their performance and QoL severely and negatively. In a study in Tehran (Iran), the effects of processed honey and royal jelly on cancer-related fatigue symptoms were examined in the patients undergoing chemotherapy, hormone therapy, and radiotherapy or chemotherapy plus radiotherapy. In the control group, pure honey was used, while the case group received processed honey and royal jelly. Patients in both groups consumed 5 ml of the supplement twice a day for 1 month. The statistical comparison of the results showed that by passing two and four weeks from the treatment with processed honey and royal jelly, fatigue severity scale was better in the case group compared to the control group.^87^ Effects of different types of honey against cancers, the potential mechanisms and corresponding references are summarized in Table 1.

**Table 1:** The potential anticancer mechanisms of different types of natural honey

**Honey type**	**Origin**	**Type of cancer/cancer cell line**	**Potential anticancer mechanism(s)**	**Reference**
TLH	Malaysia	Breast cancer induced by DMBA in rat	Induction of apoptosis, angiogenesis modulation	^28^
TLH	Malaysia	Human breast adenocarcinoma cell lines (MCF-7 and MDA-MB-231 cells) and cervical (HeLa) cancer cell line	Induction of apoptosis, *P*_53_ regulation, facilitation of antitumor effect of anticancer drugs (enhances the anticancer activity of Tamoxifen), cell cycle modulation, disruption of mitochondrial membrane potential	^3,34,38-40,68^
TLH	Malaysia	Leukemia cell lines (K562 and MV4-11), OSCC and HOS cell lines	Anti-proliferative activity, induction of apoptosis	^63,64^
Polyfloral natural honey	Iran (East Azerbaijan)	DMBA-initiated and croton oil-promoted skin carcinogenesis in mice	Antioxidant activity	^29^
Polyfloral natural honey	Iran (Khorasan)	Human renal cancer (ACHN) cell lines	Anti-proliferative activity, induction of apoptosis	^60^
Polyfloral natural honey	Iran (Zagros Mountains)	Chemotherapy-induced OM (a clinical trial)	Control of cancer-related complications	^72^
Polyfloral natural honey	Iran (Semnan)	Radiation-induced OM (a clinical trial)	Control of cancer-related complications	^73^
Processed natural honey	Iran	Cancer-related fatigue (a clinical trial)	Control of cancer-related complications	^87^
Polyfloral natural honey	India	Colon cancer cell lines (HCT 15 and HT 29), EAC model in mice	Anti-proliferative activity, induction of apoptosis, antioxidant activity	^49,65^
Polyfloral natural honey	India	Radiation-induced OM (a clinical trial)	Control of cancer-related complications	^75,76^
ACH	Pakistan	Prostate cancer (PC-3) cell line, lung cancer (NCI-H460) cell line	Anti-proliferative activity, induction of apoptosis, anti-inflammatory effect, immunomodulatory activity, *P*_53_ regulation, cell cycle arrest	^33,54,58^
ACH	Italy	Human (A375) and murine (B16-F1) melanoma cell lines	Anti-proliferative activity, cell cycle arrest	^33^
GLH	Malaysia	Colon cancer cell lines (HCT-116 and HT29), human hepatocellular carcinoma (HepG2) cells	Induction of apoptosis, anti-inflammatory effect via the NFκB pathway, facilitation of antitumor effect of anticancer drugs, modulating the expression of genes involved in the KRAS/ERK/ PI3K/AKT pathways, anti-proliferative activity, antioxidant activity, free radical scavenging effect, induced DNA damage	^45,48,52,53^
MKH	New Zealand	Murine melanoma (B16.F1), colorectal carcinoma (CT26), human breast cancer (MCF-7) cells	Anti-proliferative activity, induction of apoptosis, control of cancer-related complications (alleviating chemotherapy-induced toxicity)	^59^
Thyme honey (THH)	Cyprus	Radiation-induced oral mucositis (OM), Xerostomia-induced by chemotherapy and radiotherapy (clinical trials)	Control of cancer-related complications	^74,82^
Thyme, fir and pine honey extracts	Greek	Breast (MCF-7), endometrial (Ishikawa) and prostate (PC-3) cancer cells	Modulate estrogenic activity, prevention of cancer-related processes	^35^
Unifloral rosemary, unifloral and polyfloral heather honeys	Spain	Human hepatocellular carcinoma (HepG2) cells, human peripheral blood promyelocytic leukemia cell line (HL-60)	Antioxidant activity, free radical scavenging effect, anti-proliferative activity, induction of apoptosis	^46,62^
Natural honey	Egypt	EAC model in mice, diethyl nitrosamine-induced liver cancer in rat	Immunomodulatory activity, anti- inflammatory effect, antioxidant activity	^31,47^
Natural honey	Egypt	Human hepatocellular carcinoma (HepG2) cells	Antioxidant activity, induction of apoptosis, free radical scavenging effect, anti-proliferative activity	^3,43^
Natural honey	Egypt	Cisplatin-induced nephrotoxicity in rat	Control of cancer-related complications (nephroprotective against cisplatin-induced renal injury)	^84^
Natural honey	Egypt	Renal toxicity of cisplatin (a clinical trial)	Control of cancer-related complications (protective against cisplatin-induced renal injury)	^85^
Natural honey	USA	Cisplatin-induced nephrotoxicity in mice	Control of cancer-related complications (protects the kidney against cisplatin nephrotoxicity by suppressing inflammation and NF-κB activation)	^83^
Natural honey	Brazil	Walker 256 carcinoma cell implanted in rats	Anti-proliferative activity, induction of apoptosis	^42^
Natural honey	Japan	Human bladder cancer cell lines (RT4, T24 and 253J cells), murine bladder cancer cell line (MBT-2) and* in vivo*bladder cancer model in mice	Anti-proliferative activity	^55^
CDH	Egypt	EAC model in mice	Antioxidant activity, immunomodulatory activity	^30,32^
Jungle honey	Nigeria	Lewis Lung Carcinoma/2 (LL/2) cells	Immunomodulatory activity	^67^
Ziziphus honey	Pakistan	Radiation-induced OM (a clinical trial)	Control of cancer-related complications	^77^
Sunflower honey	Germany	Postmenopausal women with breast cancer who treated with tamoxifen (a clinical trial)	Control of cancer-related complications (decreases menopausal complaints)	^86^
Bee products mixture (royal jelly, pollen grains and honey)	Egypt	Genotoxic effects of cyclophosphamide in mice	Facilitation of antitumor effect of anticancer drugs (improved the genotoxic effects of cyclophosphamide)	^69^
Bee honey products and polyfloral honey	Croatia	Transplantable mammary carcinoma and fibrosarcoma in murine, anaplastic colon adenocarcinoma of Y59 in rat	Immunomodulatory activity, antimetastatic effects	^50,51^

Abbreviations: TLH, Tualang honey; OSCC, oral squamous cell carcinomas; HOS, human osteosarcoma; EAC, Ehrlich ascites carcinoma; ACH, Acacia honey; GLH, Gelam honey; MKH, Manuka honey; CDH, Coriander honey; OM, oral mucositis.

**Figure 3:**
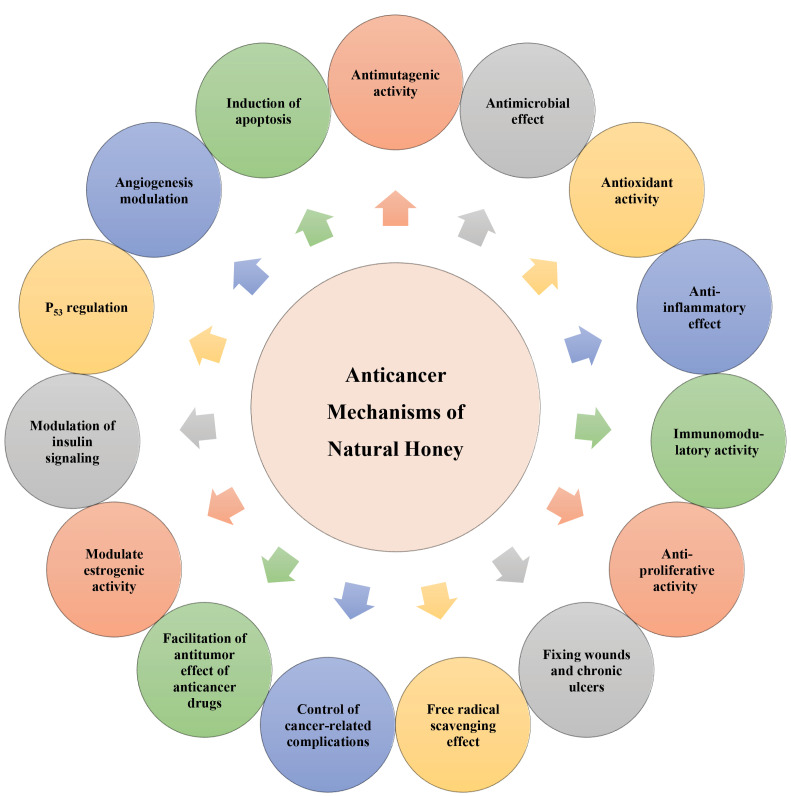
The main proposed anticancer mechanisms of natural honey.

In Figure 3, the main proposed anticancer mechanisms of natural honey are schematically presented.

### Adverse effects of honey

Natural honey is relatively free of any toxicity. At the same time, its topical application may cause a temporary stinging sensation. Although being allergic to honey is rare, it can cause some allergic reactions to bee proteins or pollen.^14^ The allergic reactions reported are ranged from cough to anaphylaxis.^88^ Tissue’s dehydration may occur by excessive use of honey, which can be compensated by saline. Theoretically, the risk of blood glucose elevation in patients with diabetes is high when honey is applied on large open wounds. However, the risk of wound botulism due to *Clostridia* spores can be minimized by gamma radiation that kills the spores without losing any antibacterial activity of honey.^14^

During production, collection or processing, honey may be contaminated by microorganisms from bees, plants, and dust.^10^ To warrant its high quality, prolong its shelf life, and maintain its freshness, honey is usually processed by heating or sterilizing. Heating leads to the formation of dangerous compounds like 5-hydroxymethyl furfural (HMF) to human health, which is not naturally present in honey. HMF is a potential carcinogenic, mutagenic, and cytotoxic agent.^16^ Honeybees are able to fly within a radius of 4 km of their hive; therefore, they have access to an area of approximately 50 km^2^. Because bees have contact with air, soil, and water, the level of heavy metals in honey may reflect the actual amount of these metals in the environment. Honey contains some potentially toxic heavy metals such as Pb, Hg, Co, Cr, As, and Cd, all of which have detrimental effects on human health.^16^

## Conclusion

Although the anti-cancer effects of various dietary compounds have been studied so far, honey has been suggested as a promising agent for the prevention and treatment of cancer. Regarding many evidences, it seems that honey and its active substances can act as anticancer compounds through various mechanisms. Although the exact and full mechanisms of these effects have not been well elucidated yet, various studies have shown how these anti-inflammatory and antioxidant functions of honey can prevent the initiation, promotion, and progression of cancer. By affecting on multiple targets, honey interferes with cancer cell’s signaling pathways, including apoptosis induction; mitochondrial pathway activation; cell cycle arrest; insulin signaling and oxidative stress modulation; inflammation amelioration; inhibition of angiogenesis and cell proliferation; immune cells and TNF-*α*, IL-1β, IFN-*γ*, and *p53* stimulation; and lipoprotein oxidation, IL-1, IL-10, COX-2, lipoxygenases, and prostaglandin E_2_ inhibition. In addition, honey improves the activity of anti-neoplastic agents and the QoL in patients undergoing chemotherapy. Further studies should be conducted to confirm the anticancer function of honey before recommending its usage in clinical interventions for patients with cancer.

## Ethical Issues

Not applicable.

## Conflict of Interest

The authors declare no conflict of interest in this study.
